# The Potential Contribution of Mass Treatment to the Control of *Plasmodium falciparum* Malaria

**DOI:** 10.1371/journal.pone.0020179

**Published:** 2011-05-24

**Authors:** Lucy C. Okell, Jamie T. Griffin, Immo Kleinschmidt, T. Déirdre Hollingsworth, Thomas S. Churcher, Michael J. White, Teun Bousema, Chris J. Drakeley, Azra C. Ghani

**Affiliations:** 1 Department of Infectious Disease Epidemiology, MRC Centre for Outbreak Analysis and Modeling, Imperial College London, London, United Kingdom; 2 Department of Infectious Disease Epidemiology, London School of Hygiene and Tropical Medicine, London, United Kingdom; 3 Department of Immunology and Infection, London School of Hygiene and Tropical Medicine, London, United Kingdom; Mahidol University, Thailand

## Abstract

Mass treatment as a means to reducing *P. falciparum* malaria transmission was used during the first global malaria eradication campaign and is increasingly being considered for current control programmes. We used a previously developed mathematical transmission model to explore both the short and long-term impact of possible mass treatment strategies in different scenarios of endemic transmission. Mass treatment is predicted to provide a longer-term benefit in areas with lower malaria transmission, with reduced transmission levels for at least 2 years after mass treatment is ended in a scenario where the baseline slide-prevalence is 5%, compared to less than one year in a scenario with baseline slide-prevalence at 50%. However, repeated annual mass treatment at 80% coverage could achieve around 25% reduction in infectious bites in moderate-to-high transmission settings if sustained. Using vector control could reduce transmission to levels at which mass treatment has a longer-term impact. In a limited number of settings (which have isolated transmission in small populations of 1000–10,000 with low-to-medium levels of baseline transmission) we find that five closely spaced rounds of mass treatment combined with vector control could make at least temporary elimination a feasible goal. We also estimate the effects of using gametocytocidal treatments such as primaquine and of restricting treatment to parasite-positive individuals. In conclusion, mass treatment needs to be repeated or combined with other interventions for long-term impact in many endemic settings. The benefits of mass treatment need to be carefully weighed against the risks of increasing drug selection pressure.

## Introduction

In the last few decades, antimalarial drugs that act against *Plasmodium falciparum* have been used primarily to avert severe morbidity and mortality. However, antimalarials have also been given to asymptomatic parasite carriers, particularly during historical malaria eradication programmes in the 1950s–1970s, with the aim of preventing onward transmission to mosquitoes and potentially interrupting transmission [1]. During the ongoing scale up of malaria interventions, a number of control agencies are reconsidering or piloting a mass treatment approach to aid transmission reductions (for example [2]). However past programmes had mixed success and were linked to increases in drug resistance [3], [4], as well as requiring a relatively high level of resources. The intervention is not currently recommended by the World Health Organization, although there is interest in further research [5], [6]. Given the potential drawbacks, it is important to better understand the extent to which this intervention could reduce transmission across different endemic settings.

Mass drug administration (MDA) involves distributing a curative regimen of antimalarials to each member of a population, regardless of the presence of parasitaemia or symptoms suggestive of malaria, while mass screening and treatment (MSAT) programmes treat only parasitaemic individuals. In theory, the malaria parasite may seem vulnerable to a mass treatment programme which targets the infectious reservoir in humans. The lifespan of malaria vectors is at most a few weeks and there is no significant animal reservoir of *falciparum* malaria. In practice, however, some individuals do not participate in mass treatment (due to refusal or health conditions that preclude antimalarial treatment, for example), and drugs may reach ∼95–98% efficacy but are not 100% efficacious even where there is no resistance [7]. Remaining parasite carriers can then be the source for re-establishment of malaria transmission, potentially rapidly. Past MDA interventions have been extensively reviewed [1]. The impact of these programmes is difficult to assess because (1) MDA was usually combined with simultaneous vector control, (2) few trials had sufficient if any control populations since most were conducted before the development of cluster-randomized trial methodology and (3) measurement of impact on transmission was frequently carried out for too short a time after the MDA. These limitations notwithstanding, most trials report at least a transient effect on malaria transmission, although in some cases this was very small or of short duration [8]. Four studies reported local elimination of malaria at least for a number of years [9], [10], [11], [12]; all of these combined MDA with indoor residual spraying. However a mass administration of pyrimethamine in Tanzania was followed shortly afterwards by the appearance of clinical resistance in the population [3]. The increased selection pressure on parasites is likely to be an important drawback of mass treatment.

Control agencies working on other infectious diseases have more recent and extensive experience with mass treatment programmes [13]. For example, large cluster-randomized trials have been carried out to assess the impact of MDA programmes on trachoma transmission and theoretical insights have been gained from mathematical modeling [14]. Mass treatment programmes for trachoma can achieve a reduced prevalence for around 2 years following a single round of treatment. However, in most places, transmission returned to pre-intervention levels over time in the absence of further intervention [15], [16].

There are several questions which need to be addressed to inform researchers and policy makers who are considering piloting mass treatment for malaria control. It would be useful to know whether mass treatment is best used during initial stages of control programmes to aim for large reductions in prevalence, or to clear remaining infections after other control measures have already reduced transmission. Screening before treatment may be preferred to reduce the number of treatments required and to prevent unnecessary risk of adverse reactions in uninfected individuals. However this would be logistically more demanding and may not have the same impact as an MDA programme. The advantage of using treatments with gametocytocidal and prophylactic effects has been discussed but the difference in impact of mass treatment between different types of antimalarials has not been formally tested. It would also be helpful to know to what extent mass treatment could have a role in elimination as part of a wider control programme, and in what settings this could be achieved. Mathematical models of mass treatment for malaria have examined the influence of transmission intensity and seasonal timing of the intervention [17], [18], [19]. One model successfully predicted the local elimination of *falciparum* malaria by 9 rounds of MDA in a specific low transmission island setting (Aneityum in Vanuatu) in combination with insecticide-treated nets [12], [20]. We use a recently published individual-based model which was developed to look at the impact of multiple interventions [21], and includes additional aspects of malaria epidemiology which have been found to be important to accurately estimate reductions in transmission, such as heterogeneity in exposure to bites in the human population [22]. Here we characterize the influence of mass treatment on malaria transmission dynamics using this model and explore the impact of different strategies for the implementation of mass treatment.

## Methods

### Transmission model

We use a previously described dynamic individual-based stochastic model [21] which captures key aspects of the *P. falciparum* lifecycle and its transmission between human and mosquito populations. The baseline model and its parameters in the absence of control interventions have been validated by statistical fitting to data from a wide variety of endemic settings. Here we summarize key aspects of the model. Parameters are as previously described, except those shown in Table 1 which have been added or modified to describe the mass treatment intervention in more detail.

**Table 1:** Key parameters used in the model with references.

*Definitions & units*	*Estimates (data/literature)*	*Values used in model*
**Drug efficacy** (% treated individuals with no parasitological treatment failure, all drug types)	ACT 95–98% [7]	95
**Duration of gametocytaemia in treated infection, days**		
non-artemisinins	55.6 [43]	55.6
ACT	13.4 [43]	13.4
ACT-PQ	3.0 [43]	3.0
**Duration of inhibitory antimalarial blood concentration, days**		
‘Short-acting’ drug	7 SP N51I, S108N mutant, partially resistant [46]	10
	8.5–12.4 lumefantrine [47]	
‘Long acting drug’	30 SP, low prevalence of resistant strains [48]	30
**% reduction in average infectiousness following treatment compared to state occupied prior to treatment**		
non-artemisinins	70 based on [42]	70
ACT, ACT-PQ	80.6 [49]	80.6
**Pregnancy prevalence**		
Among women aged 15–45	14.2% based on [50]	-
Among the population aged 15–45	-	7.1%
**Correlation in individual participation between repeated rounds of MDA**	-	0.5

Parameters listed here are those which are additional or different from the previously published model [21].

Humans are categorized into one of 6 states: susceptible and uninfected *S*; symptomatic and infectious *D*; asymptomatic and infectious *A*; infectious and undetectable by standard microscopy *U*; treated and infectious *T*; uninfected and protected by antimalarial prophylaxis *P*. Newly infected humans develop patent parasitaemia after a time delay representing the liver stage of the parasite. A proportion of those infected develop symptoms and may be successfully treated with an antimalarial, with a subsequent period of protection against new infections of a duration dependent on the half life of the antimalarial. In the absence of treatment or following treatment which does not fully clear parasitaemia, symptomatic individuals progress to asymptomatic infection. Asymptomatic infections in state *A* progress to the subpatent state *U* (see below for more detail). We allow for superinfection in those who are infected. Based on parameter fitting we assume that the symptomatic, untreated state *D* results in the highest probability of human-to-mosquito transmission, after a fixed delay period allowing for the development time of gametocytes. Asymptomatic cases *A* are approximately 3-fold less likely to infect a biting mosquito, and the subpatent stage *U* approximately 17-fold less likely [21]. The infectivity of treated infections before parasite clearance depends on the antimalarial used and the state of infection prior to treatment (see below for details). Among symptomatic cases receiving treatment we assume a constant 20% coverage of artemisinin-combination therapies (ACTs) and 80% coverage of non-ACT treatments (such as sulphadoxine-pyrimethamine) [23]. The non-gametocytocidal treatments are assumed to resolve symptoms but to be only 60% efficacious at clearing parasites, due to resistance, while ACTs are assumed to be 95% efficacious. Treatment failures enter the asymptomatic *A* state.

The human population model is fully age-structured. It allows for three types of immunity: infection-blocking which reduces the probability of becoming infected following a bite from an infectious mosquito; clinical immunity which reduces the probability of developing symptoms upon infection, and blood-stage immunity which speeds recovery from the asymptomatic, patent state. We also allow for heterogeneous exposure among humans to mosquito bites. The human and vector populations are assumed to be static, with no possibility for reintroduction of infection by migration. We assume a human population size of at least 10,000 and average over a minimum of 10 stochastic realizations, unless otherwise indicated. Simulations using this population size closely approximate results from simulations of larger populations (confirmed using a population size of 250,000, results not shown).

As previously described, the vector population is modeled with a seasonally-forced Susceptible-Exposed-Infectious (SEI) structure. Vectors are assumed to have characteristics of *Anopheles gambiae s.s.* except in simulations comparing the model to trial data (see below).

### Interventions

As previously described, we assume an MDA programme treats a percentage of individuals in the population regardless of infection status. For simplicity, treatment is assumed to occur in all individuals instantaneously. Those who are uninfected at the time of mass treatment enter the protected state *P* for a time dependent on the half life of the drug, with a probability dependent on the efficacy of the drug (defined as the probability of full parasite clearance). Infected individuals who are successfully treated by the MDA progress to the treated, infected state *T* and clear parasites more quickly, and may also enter the protected state after recovery depending on the half life of the drug. An MSAT programme is implemented in the same way in the model except that it is given to individuals selected by screening tests. If PCR is used as the screening tool, those in the infected *A*, *D* and *U* states are treated, while if microscopy is used, only those in the *D* and *A* states are treated. The model was previously fitted to detailed data available on the prevalence of microscopy-positive infection versus PCR-positive infection by age, with PCR detection assumed to be the gold-standard method [21]. These data suggest a sensitivity of microscopy around 50–75%. Under these model assumptions, the most infectious individuals (*D* and *A*) are identified and treated during an MSAT programme using microscopy (Table 1). In sensitivity analyses, we also modeled MSAT assuming the screening test would have 75% or 50% sensitivity to detected infected individuals regardless of whether in the *D*, *A*, or *U* states, since the sensitivity of microscopy can vary widely [24].

We contrast the impact of using antimalarials with different properties for mass treatment: non-gametocytocidal antimalarials such as sulphadoxine-pyrimethamine-amodiaquine (SP-AQ) versus gametocytocidal antimalarials such as combinations containing artemisinin (ACT) or artemisinin and primaquine (PQ); and short-acting antimalarials such as lumefantrine versus long-acting antimalarials such as SP or piperaquine. Sources for parameters describing antimalarial effects are given in Table 1 (see Text S1 for further detail). We model a constant percentage reduction in infectivity of infected individuals by a given treatment compared to the infectivity of the state occupied in the previous time step. We assumed all antimalarials used for mass treatment would successfully clear parasites from 95% of infected cases to give a fair comparison of their pharmacodynamic properties, although this may not be the case particularly for non-ACT treatments such as SP-AQ in many endemic areas.

As previously [21] we considered the likelihood that when multiple rounds of mass treatment are carried out, the same individuals may be repeatedly less likely to participate, which could reduce the impact of the intervention. The correlation in the probability of participating in successive rounds of the intervention can be between 0 (mass treatment is distributed randomly in the population) and 1 (the same individuals always participate or never participate in mass treatment). Modern mass treatment programmes may not include pregnant women for safety reasons. For these simulations we assumed that women of child-bearing age would be tested for pregnancy and excluded from treatment upon a positive test result. We estimated prevalence of pregnancy based on fertility data from 25 Sub-Saharan African countries (see Text S1). For simplicity we did not assume any difference in susceptibility of infection among pregnant women compared to other adults. Since mass treatment would rarely be used as a single intervention, we also explored the impact of a programme in which vector control is used simultaneously (details are given in Text S1 and previous publication [21]). Further details of the model methods are given in Text S1.

### Outcomes

We assess the impact of mass treatment over time on both the EIR and prevalence of infection detectable by microscopy of blood slides. We report the cumulative EIR or prevalence reduction which is the sum of the reduction each day compared to pre-intervention levels over the specified number of days. We also examined the probability of local elimination in the model in populations of different sizes from 500 stochastic realizations for each scenario to identify the proportion in which elimination occurred. For results where we were interested in the average outcome in a population over the long term we also considered as an outcome a pre-elimination stage, which was defined as a scenario where slide-prevalence <0.1% for at least 50 days. The pre-elimination outcome was used because the outcome of full elimination in large populations in the long term is sensitive to the assumed time-scales of decline of immunity following reduced exposure, which are uncertain [25] as well as large-scale spatial heterogeneity in transmission which is not currently considered in the model.

### Trial data

We compared our model output with results of a field trial of MDA which took place in Burkina Faso in 1960–1961 [26]. 5 villages (1890 people) received 8 rounds of MDA at 28 day intervals, 3 villages (2560 people) received MDA every fortnight for a total of 15 rounds and there were 6 control villages with no intervention. Within villages, individuals were randomized to receive chloroquine-primaquine or amodiaquine-primaquine. We fitted our model to the measured seasonal entomological inoculation rate (EIR) using maximum likelihood and compared our output to the slide-prevalence in 2–10 year olds before, during and after the intervention period (see Text S1 for full details).

## Results

### Rebound in transmission following mass treatment & comparison with trial data

**Figure 1:**
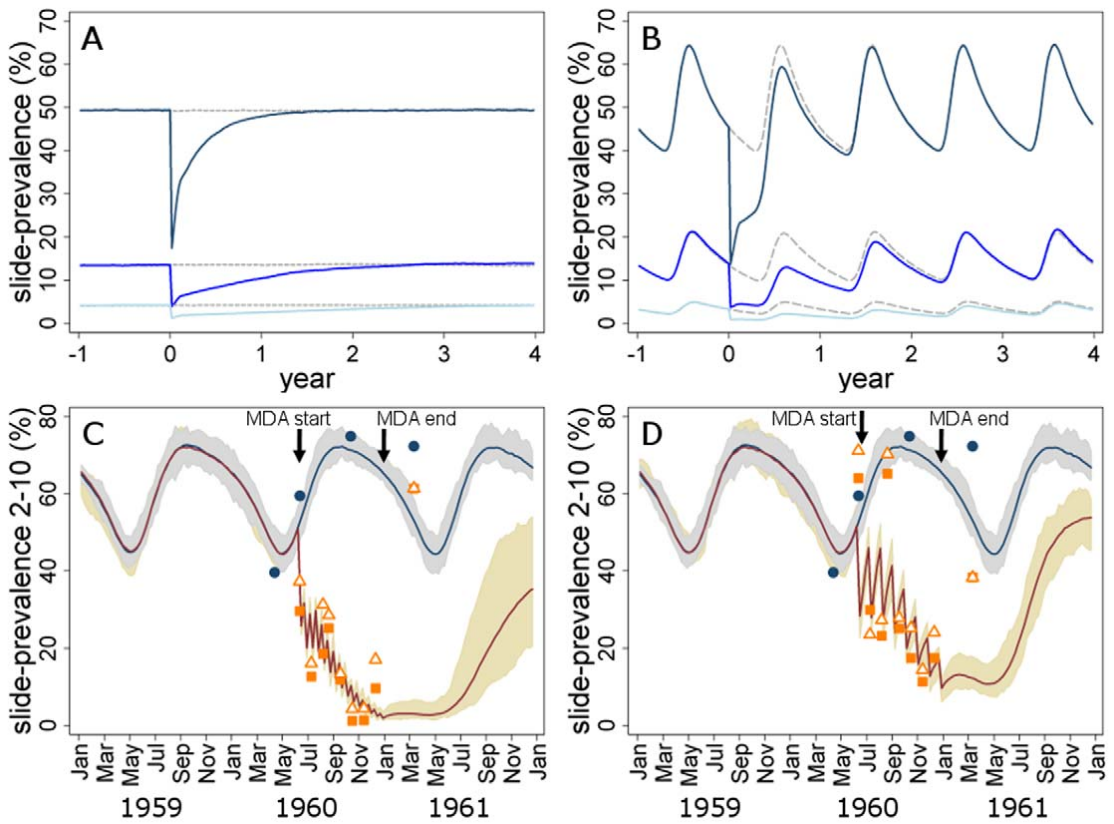
Rebound in transmission following mass treatment & comparison with trial data. (A) & (B) Typical MDA model output over time in scenarios of (A) non-seasonal and (B) seasonal transmission with different baseline transmission intensities: high: baseline average annual slide-prevalence = 50% (dark blue); medium: baseline slide-prevalence = 15% (mid-blue); low: baseline slide-prevalence = 5% (light blue). A single round of MDA is carried out at year 0 at 80% coverage (actual coverage is lower due to exclusion of pregnant women). Gray dashed lines indicate predicted prevalence in the absence of MDA. (C) & (D) Comparison of model predictions with trial data [26]: slide-prevalence in 2–10 year olds in control villages and intervention villages with MDA carried out (C) every 14 days or (D) every 28 days. Blue line = average model-predicted values in control villages; dark red line = model predicted value in MDA villages; shaded areas = range of 20 simulations of control and MDA prevalence; blue circles = prevalence data in control villages; orange triangles = prevalence data from chloroquine-treated individuals in MDA villages; orange squares = prevalence data from amodiaquine-treated individuals in MDA villages.

The predicted impact of a MDA programme with one round of treatment and no screening criteria is shown (Figures 1A & 1B, Table 2). Unless otherwise indicated, we assume in all results that an ACT with a short half life is used for mass treatment and that coverage is 80%. Immediately following MDA there is a dramatic drop in slide-prevalence due to successful cure of infection. However transmission of the parasite can on average persist in the population in the scenarios shown, given that mosquitoes remain infected, treated cases transmit to mosquitoes for some time after treatment, coverage is not 100%, and treatment efficacy is not 100% even in the absence of drug resistant parasites (e.g. due to incomplete drug absorption). The prevalence of infection is therefore predicted to rise again following MDA until it eventually reaches the pre-intervention level, as found in previous analyses [18]. The reason for this is that the key factors which determine local transmission intensity and therefore prevalence of infection are the local density of mosquitoes, their rate of biting humans, and the rate at which infected humans clear parasitaemia. Once inhibitory blood drug levels decline in those participating in the mass treatment none of these factors have been changed permanently, in the absence of any other control interventions. Figures 1A–1B and Table 2 show the average predictions of impact in a large population. In a later section, we consider the potential for temporary local elimination in smaller populations due to chance events.

**Table 2:** Short-term and long-term impact of a single round of MDA.

*Outcome & setting*	*Absolute number*	*% of total*
**Cases cured by direct effect of MDA per 100 treated**		
( = prevalence×coverage (deducting pregnant women)×drug efficacy)		
High transmission	35.3	70.6%
Medium transmission	10.6	70.6%
Low transmission	3.5	70.6%
**Duration of impact on transmission intensity**		
(months to return to 90% of baseline slide-prevalence)		
High transmission	7	-
Medium transmission	19	-
Low transmission	35	-
**Cumulative EIR reduction over the 2 years following MDA**		
(infectious bites averted per person)		
High transmission	26.7	9%
Medium transmission	1.2	22%
Low transmission	0.3	35%
**Cumulative prevalence reduction over the 2 years following MDA**		
(days of infection averted per person)		
High transmission	37	10%
Medium transmission	27	27%
Low transmission	13	41%

An MDA programme is simulated which uses a short-acting ACT with 80% coverage (before exclusion of pregnant women) in 3 areas with different initial transmission levels: high (baseline slide-prevalence = 50%), medium (baseline slide-prevalence = 15%) and low (baseline slide-prevalence = 5%). Results are shown for scenarios which have no seasonal variation in transmission (as in Figure 1A).

The impact of MDA in the short and long term depends on the initial level of transmission in an area (Figures 1A & 1B, Table 2). In the short term, if 80% of randomly-selected individuals are treated in any given population, there will naturally be higher absolute numbers of infected individuals cured directly by the MDA if 50% of people are infected than if 5% of people are infected. However the long term duration of MDA impact is predicted to be much longer in low transmission settings, as found in previous analyses [14], [18]. In the low transmission scenario (baseline slide-prevalence = 5%) without seasonal variation in transmission, the prevalence of infection takes around 3 years to return to baseline. By contrast in areas of high transmission, frequent vector biting means that parasites surviving after mass treatment are spread rapidly through the population. We find that in a high transmission scenario of 50% baseline slide-prevalence, levels of infection return close to baseline within 7 months after a single round of MDA (Figure 1A). The cumulative impact of the intervention over time in terms of the absolute number of infectious bites averted per person is highest in the high transmission scenario, where a round of MDA is estimated to prevent 27 infectious bites per person compared to 0.3 infectious bites per person in the low transmission scenario over the 2 years following the interventions (Table 2). However in the high transmission scenario the reduction amounts to only 9% of total infectious bites received over the 2 years, while in the low transmission scenario 35% of infectious bites are prevented (Table 2). The reduction in the number of days spent infected shows a similar pattern. The same rebound effects can be seen in seasonal transmission settings (Figure 1B).

The model was able to reproduce the results of a previously published MDA trial [26] reasonably well for the time period in which EIR data were available although the predictions varied according to the model assumptions (Figures 1C and 1D). Prevalence was lower in some intervention villages before the start of MDA (Figure 1C), although it is not clear if this resulted from a long-term difference in transmission intensity between control and intervention villages or chance variation. During MDA, prevalence reduced substantially in both intervention groups but 3 months after the end of MDA, prevalence rose rapidly and reached a level close to that in control groups. Based on the reported EIR, the model prediction of slide-prevalence was higher than observed in the data in all trial arms (see Text S1). The model was previously fitted to a large number of paired EIR and prevalence data points [21] but there is variability in the relationship between these measures across age groups and geographic sites and EIR measurements are imprecise. Therefore we also ran simulations in which we reduced average annual mosquito densities (keeping the seasonal pattern and the ratio of *An. gambiae* to *An. funestus* constant) to match the observed prevalence in the control villages. This matched the intervention group prevalence data better at the baseline and during the MDA intervention, although worsened the fit at the follow up measure (see Text S1). Assuming a lower efficacy of treatment further improved the model prediction (Figures 1C & 1D), except for some outlying values in the MDA group who were treated every 28 days (Figure 1D). After the intervention was stopped, prevalence rose more quickly in the data than was predicted by the model. However EIR data were lacking for this time period and control prevalence was also higher than predicted based on the EIR at the same time during the year before, suggesting that the annual EIR in the second season may have been higher than in the first season. Full details and sensitivity analysis are given in Text S1.

### Choice of MDA strategy

#### Timing

**Figure 2:**
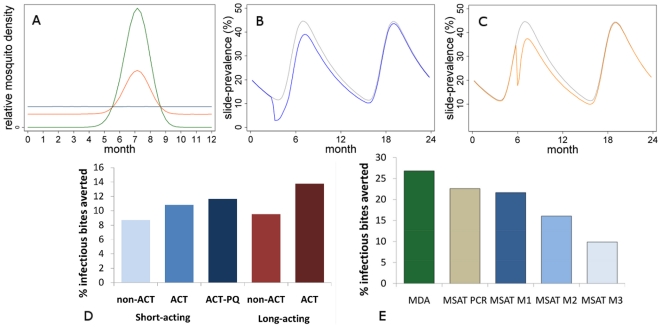
Strategy options for mass treatment. (A) Three scenarios of seasonal mosquito densities simulated by the model: green line = highly seasonal: 96% of infectious bites occur in the peak 3 months; red line = moderately seasonal: 50% of infectious bites occur in the peak 3 months; blue line = not seasonal (for comparison). (B)&(C) Example simulations showing the impact of MDA on slide-prevalence in a scenario of highly seasonal transmission if carried out (B) blue line: prior to the rise in mosquito densities (month 3 in this simulation) as shown in (A), versus (C) orange line: just prior to the peak of the transmission season (month 6 in this simulation). Baseline slide-prevalence in the absence of MDA is shown in gray for comparison. (D)&(E) Model-estimated cumulative % infectious bites averted per person after 1 round of mass treatment over the following 2 years (D) comparing different antimalarial types and (E) comparing different screening criteria used to allocate treatment, in a scenario of moderate transmission intensity (baseline slide-prevalence = 15%) with moderate seasonal variation (as in Figure 2A). Mass treatment is carried out prior to the high transmission season. ‘Short-acting’ = 10 days prophylaxis, ‘long-acting’ = 30 days prophylaxis. MDA = no screening; MSAT PCR = PCR-positive individuals are treated; MSAT M = microscopy-positive individuals are treated; MSAT M1 = microscopy detects all those in the more infectious *A* and *D* states in the model and not those in the *U* state (microscopy sensitivity = 58% in the scenario shown); MSAT M2 = microscopy sensitivity is 75% for infected individuals regardless of infection state (*D*, *A* or *U*); MSAT M3 = microscopy sensitivity is 50% for infected individuals regardless of infection state (*D*, *A* or *U*).

To explore optimal timing of MDA in a seasonal setting we considered scenarios with different degrees of seasonality in transmission (Figure 2A). We find that the greatest cumulative impact on EIR is generally achieved when MDA is carried out prior to the rise in EIR at the start of the higher transmission season, in line with previous analyses [18] (Figure 2B). The worst time to carry out an MDA is predicted to be at the peak of the transmission season or just beforehand (Figure 2C), since high transmission rates at this time allow the parasite prevalence levels to recover quickly. The cumulative number of infectious bites prevented per person in the two years following MDA in our moderate transmission scenario is 2 if MDA is done at the optimum time (Figure 2B) and 0.1 if done at the least effective time (Figure 2C).

#### Choice of antimalarial: gametocytocidal and long-acting antimalarials

We explored the use of antimalarials with different properties for MDA (Figure 2D) in scenarios of high, medium and low transmission intensity (as in Figures 1A & 1B). We estimate that using a short-acting ACT for MDA could produce a 24–26% higher reduction in EIR than a short-acting non-gametocytocidal antimalarial, while a short-acting ACT-primaquine combination could achieve a 34–35% higher impact. We also find that a long-acting antimalarial regimen which provided prophylaxis would give a small advantage over a short-acting antimalarial (a 2–11% higher cumulative EIR reduction, with greater effect in the medium and high transmission scenarios). A long-acting ACT is predicted to give the highest impact, with a 47–63% higher cumulative reduction in EIR than a short-acting non-gametocytocidal antimalarial. However long-acting antimalarials may enhance the development of resistance (see discussion).

#### Mass Screen and Treat (MSAT) versus Mass Drug Administration (MDA)

MSAT is predicted to have a slightly lower impact than MDA (Figure 2E). The cumulative reduction in infectious bites per person over the year following a single round of MSAT with PCR as the screening tool was estimated to be 84% of the reduction achieved by MDA. Using microscopy as a screening tool during MSAT can further reduce the predicted impact depending on the assumed sensitivity of microscopy. If all those in the more infectious states *A* and *D* were detected and treated, the transmission reduction was 81% of that achieved by MDA, only slightly lower than with a programme using PCR as a screening tool which also treats those in the subpatent *U* state. The lower impact of MSAT compared to MDA is explained mostly by the lack of prophylaxis in screen-negative individuals rather than by missing subpatent infections under these model assumptions. However if microscopy detected 75% or 50% of all infected individuals regardless of infection state, the impact was 60% or 37% of that achieved by MDA, respectively.

### Potential for local elimination by mass treatment

**Figure 3:**
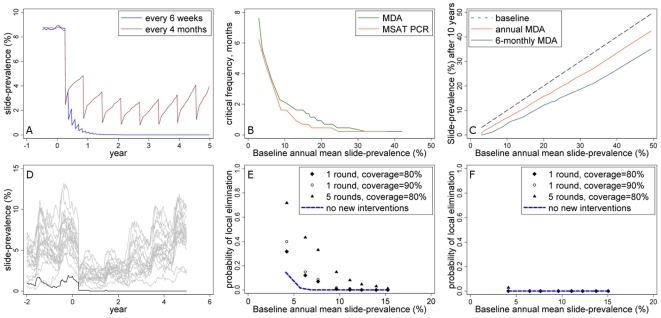
Potential for elimination by mass treatment. (A) Example simulation of a mass treatment programme in a non-seasonal scenario where multiple rounds of MDA are carried out, and the interval in between treatment rounds is less (every 4 months) or more (every 6 weeks) than the critical interval required to achieve <0.1% slide prevalence. (B) Model-estimated minimum frequency of mass treatment required in a large population to bring slide-prevalence to 0.1% or less for at least 50 consecutive days in non-seasonal settings: MDA versus MSAT with PCR as a screening tool. (C) Model-estimated annual mean slide-prevalence after 10 years of MDA repeated every year or every 6 months, according to baseline slide-prevalence prior to intervention. Moderate seasonality is assumed (see Figure 2A). D) example stochastic simulations of transmission over time assuming a small human population (n = 1000) with 1 round of MDA before the peak transmission season in year 1. A single simulation in which MDA succeeds in eliminating infection locally is shown in black. E) and F) Model-estimated probability of local elimination at different transmission intensities in a population of (E) 1000 or (F) 10,000, following MDA of different intensities. Results are based on 500 stochastic realizations per plotted point. Moderate seasonality is assumed (see Figure 2A). All simulations in this figure assume a low level of vector control at baseline which is maintained over time (the baseline slide-prevalence shown is in the presence of vector control).

We investigated what intensity of mass treatment programme would be required for elimination of infection in the simulated population. For all results presented in this section we assumed a low level of existing vector control prior to mass treatment that had reduced slide-prevalence of infection by 20–30% from its initial level (see Text S1). We then assume that this level of vector control is either maintained or scaled up. The relationship between the frequency at which mass treatment is repeated and the potential for control and elimination of infection in large populations has been previously characterized in relation to trachoma control [14]. If MDA rounds are repeated indefinitely, and each successive round of MDA can be carried out before the prevalence of infection has recovered following the previous MDA round, the parasite could theoretically be eliminated (Figure 3A). The maximum time interval between successive rounds of treatment so that slide-prevalence on average reaches a pre-elimination threshold of 0.1% is called the critical interval. The critical interval depends on speed of resurgence in transmission and therefore the initial transmission intensity (Figure 3B). Where slide-prevalence of infection is low (<5%), six-monthly rounds of MDA or MSAT using PCR screening are predicted to be sufficient to bring prevalence to the pre-elimination threshold (Figure 3B). In medium-to-high transmission settings, treatment would need to be highly frequent to achieve this target with either strategy. These results in medium-to-high transmission settings are also sensitive to the assumed level of repeated non-participation: if a sufficiently large proportion of the population never participated in mass treatment then however frequently mass treatment was carried out it would not be sufficient to reduce prevalence to zero. In medium-to-high transmission settings, less frequent rounds of mass treatment could sustain an appreciable impact on transmission if they were ongoing (Figure 3C). For example, annual MDA is estimated to lower mean EIR by 20% where baseline slide-prevalence is 40%, while six-monthly treatment rounds could achieve a 30% reduction in the same scenario.

As well as examining average outcomes in large populations, we explored chance elimination on a local scale in populations of different sizes using multiple stochastic simulations (example shown in Figure 3D). However, this outcome applies only to specific scenarios in the short-term since (1) we do not consider immigration of new infections into our human or mosquito populations and (2) elimination is highly dependent on the assumed population size (e.g. see [27]).

In small populations where malaria transmission has already been brought to a low level, a single round of mass treatment could appreciably raise the probability of elimination. For example in settings with 4% slide-prevalence, we find that the probability of chance parasite extinction in a human population of 1000 rises from 15% without any new interventions to 32% or 40% with an MDA coverage of 80% or 90%, respectively (Figure 3E). However elimination probabilities are strongly dependent on population size and initial transmission intensity, so that with a larger population size (n = 10,000, Figure 3F) or higher starting transmission level, one treatment round becomes highly unlikely to eliminate infection. An intense attack on transmission using 5 rounds of fortnightly MDA boosts the chance of local elimination, with the probability rising to >30% for settings of <8% slide-prevalence and a population size of 1000, however it remains close to zero in a larger population of 10,000 (Figure 3F).

**Figure 4:**
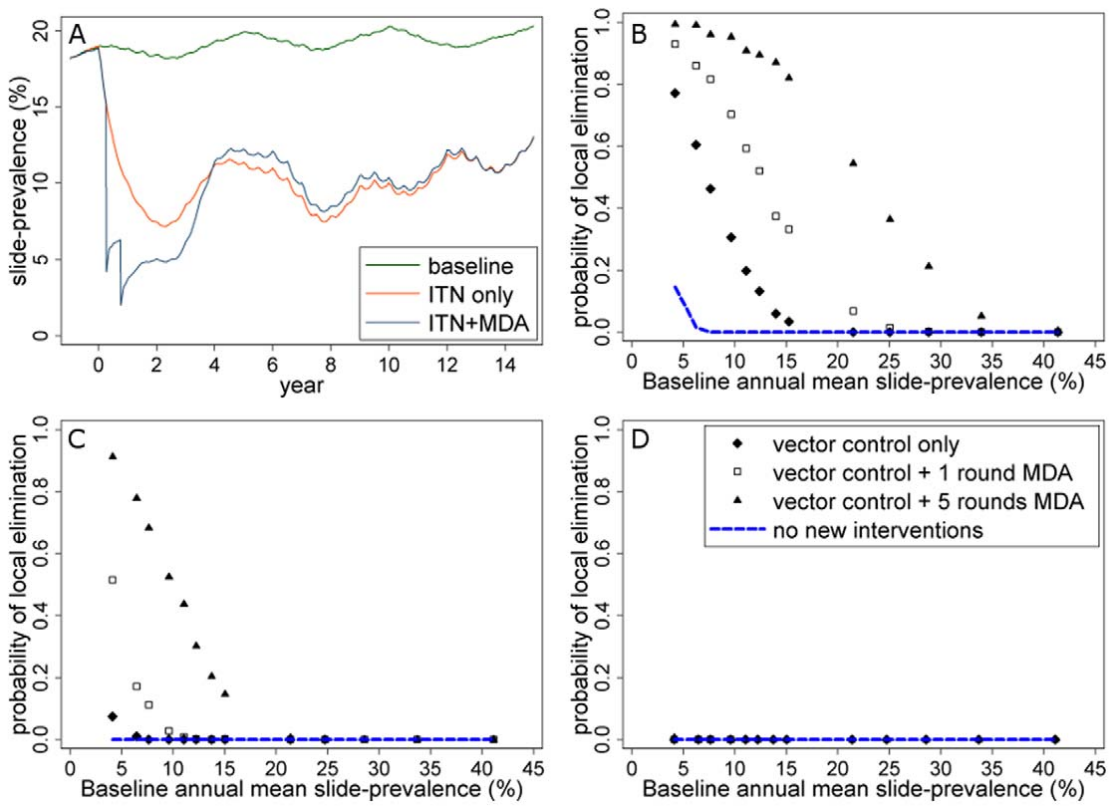
Model-estimated combined impact of MDA and vector control and the potential for elimination. (A) Vector control is scaled up in year 0, with or without 2 rounds of MDA during the first year. B), C) & D) Probabilities of local elimination in an isolated population of (B) 1000, (C) 10,000 or (D) 500,000 using vector control alone or in combination with MDA. Transmission is moderately seasonal and MDA is given prior to the transmission season.

Scaling up vector control together with MDA raises the probability of elimination above what would be achieved with either strategy alone. We assume that a scaled-up vector control programme would approximately halve slide-prevalence of infection 2 years after its introduction in most scenarios when used alone (simulations suggest this could be achieved by raising insecticide-treated net coverage to 80%, see Text S1). MDA can speed up the reduction in slide-prevalence (Figure 4A) and while it does not in the long-term produce a lower prevalence than would be achieved by vector control alone, prevalence can be brought to low levels for a period of time which raises the chance of elimination. For example in a population of 1000, estimated probabilities of elimination of >40% could be achieved in settings of up to 25% baseline slide-prevalence by 5 rounds of MDA together with simultaneous scale-up of vector control (Figure 4B). However in a larger population of 10,000, the probabilities using the same strategies would be considerably lower (Figure 4C) and would approach zero in a population of 500,000 (Figure 4D).

## Discussion

Our simulations suggest that given a favorable set of circumstances, mass treatment has the potential to be a valuable part of a malaria control programme. Specifically, where transmission rates are relatively low (<5% slide prevalence), a single round of mass treatment could lower transmission for 2 years or more. Seasonal variation in transmission can be exploited to maximize impact. Mass treatment could also appreciably increase the chance of local elimination of infection when combined with vector control in a small isolated population (for example a few thousand individuals). Such situations may occur for example on islands, or in hypoendemic countries where transmission is concentrated in small pockets. With the recent reductions in transmission that have been observed in parts of Africa [28], such settings may become increasingly common.

However, in other endemic scenarios we find that the very high transmission rates of *P. falciparum* mean that the effect of mass treatment is likely to be short-lived after the intervention is discontinued, in agreement with previous modeling work [18]. Data from MDA field trials indicated an even more rapid post-intervention increase in transmission than predicted by our simulations [26]. Therefore our estimates of the duration of impact may be optimistic although temporal variation in EIR could also account for the discrepancies between the trial data and our predictions. The basic case reproductive number R_0_ of *P. falciparum* malaria (defined as the average number of secondary cases arising in a susceptible population as a result of a single human case over the course of their malaria infection) can be over 100 in endemic areas of Africa when conditions are favourable for vectors [29]. This is considerably higher than other tropical infectious diseases for which mass treatment is a common intervention, for example trachoma (R_0_ estimate = 3 or less [30]) and schistosomiasis (R_0_ estimate = 4–5 [31]. In such areas, there is likely to be less value in a one-off mass treatment intervention. However if resources are available to repeat the intervention, reductions in EIR of around 25% could be achieved by annual MDA even in higher transmission settings (baseline slide-prevalence between 10%–30%). Although such an intervention could be resource-intensive, there may be potential to combine mass treatment for malaria with mass treatment programmes for other infections where these are in operation, such as schistosomiasis and trachoma [13], provided the timing of a combined MDA has a good impact on seasonal transmission.

Mass treatment can furthermore speed up the reduction in prevalence achieved by vector control. In a limited number of low-to-medium transmission settings with small population sizes which are isolated from other endemic areas, it may be appropriate to consider a short term intense attack on the parasite population using a number of closely spaced mass treatment rounds accompanied by vector control to aim for local elimination. However such areas would remain highly receptive to imported parasites once the vector population recovered. It would be important to prevent incoming infections by screening and treatment of visitors. Such a strategy has proved successful in Aneityum in Vanuatu, where malaria was eliminated from a small island population (n = ∼700) by scaling up insecticide-treated nets and using 9 rounds of MDA [12].

We demonstrate that MSAT using microscopy as a screening tool may have a lower impact than MDA, but our results are highly sensitive to the assumed infectivity of individuals who test microscopy-negative despite having parasitaemia. A limited number of studies suggest infectivity among these individuals is low [32], however submicroscopic infections can be very common [33] and additional studies on their contribution to the infectious reservoir would be valuable for assessing the potential impact of MSAT. We used the scenario of microscopy testing because detailed data were available on microscopy versus PCR prevalence by age [21]. We did not have the equivalent data for rapid diagnostic tests which are more likely to be used in the field. Rapid diagnostic tests may have a lower sensitivity than microscopy for detecting asymptomatic infections [34].

Our analysis finds that gametocytocidal treatments could be highly beneficial for mass treatment, particularly when combined with a long-acting drug. However, the risk of adverse reactions would need to be carefully considered, particularly for primaquine [35]. Furthermore, mass treatment programmes could increase drug pressure for the parasite population since asymptomatic and non-infected individuals are administered with a full curative dose of antimalarials that they otherwise would not have received. This increases the chance of malaria parasites encountering sub-therapeutic levels of long-acting drugs [36]. Ideally, antimalarials used in any mass treatment programmes would not be regularly used as first or second line treatments for symptomatic cases, nor would be likely to be components of future treatment regimens. It is possible that under certain conditions, specifically if transmission is very low, if nearly all malaria infections are symptomatic, and there is a high coverage of case management with the recommended treatment, mass treatment with the same drug may not add greatly to the selection pressure already in place as long as good adherence to the full treatment regimen can be achieved. However this requires confirmation through further field-based and theoretical studies, and in many endemic areas it would currently be challenging to achieve such conditions. Using MSAT rather than MDA may reduce selection pressure slightly, by reducing the number of individuals who have residual drug in their blood after the intervention has ended. [37].

Using our model, we were able to simulate the impact of multiple rounds of MDA in a published field trial reasonably well. However the rise in prevalence 3 months after the end of the intervention occurred more quickly than we estimated. This may be because we only had EIR data during the period of the MDA intervention, and it is likely that mosquito densities and the resulting transmission levels were higher in the subsequent three months compared to the previous year based on the control group prevalence. Alternatively the underestimation may be a limitation of our model structure. For example we do not allow for ‘rebound’ effects whereby asymptomatic cases who are treated lose short-term immunity (premunition), making them more susceptible to reinfection. Higher infectivity to mosquitoes during the early stages of infection in humans would also speed up the rise in prevalence following MDA. We identified only one other published MDA trial in the literature which had control groups and did not combine MDA with interventions against the vector [38]. Although we did not use this study for validation, it reported a similar swift rise in transmission after the MDA intervention finished. After one round of MDA using SP-artesunate, the number of clinical attacks in the villages receiving MDA remained lower for only one month after the intervention. However, again the EIR at the time was uncertain and there were strong seasonal dynamics. It would be informative for further trials to be done in lower transmission areas with follow up of the study populations and control groups for several months after the end of mass treatment.

One limitation of the analysis presented here is that the model does not include spatial structure. We assume that a single mosquito population interacts with a single human population, whereas over larger populations it is likely that individual mosquitoes bite only on humans within a relatively short distance. Movement of people may reintroduce infection in the area where MDA is undertaken. In general our results are more pessimistic about the ability of mass treatment to achieve elimination at a given initial transmission intensity than those generated by previous models [18], [39]. This is likely to be due in part to the inclusion of heterogeneous exposure of the human population to vector biting, which makes it more difficult to eliminate infection through establishment of ‘hotspots’ of transmission [22] and is an important feature of malaria epidemiology in the field [40], [41]. Furthermore, we assumed that some individuals were repeatedly less likely to participate in the intervention than others, therefore reducing its impact. If there were some individuals who never participated at all then the estimated impact of the intervention and probabilities of elimination would be further reduced. Our assumption of simultaneous treatment of the population which may not be realistic. A staggered treatment distribution could reduce the estimated effect as infection could be maintained in parts of the population at any given time. As with any modeling analysis, there are uncertainties in the parameters used which can affect the conclusions. In particular, the duration and level of infectivity of those who are treated relative to those with untreated infection has only rarely been measured in the same study [42], but is an important predictor of mass treatment effects. Our estimates of the duration of infectivity are based on the duration of gametocytaemia after treatment [43]; however this may overestimate the corresponding infectivity to mosquitoes which is not well characterized [44] and infectivity may decrease over time [45]. In particular our estimate of the duration of infectivity after non-ACT treatment of 55 days is relatively long, however we only assumed this duration for the result shown in Figure 2D. For all other simulations except of the historical trial (figures 1C and 1D), we assumed use of ACT treatment with an estimated duration of 13.4 days infectivity and an 80% reduction in transmission to mosquitoes. If these are overestimates, then our results may be pessimistic about mass treatment impact, however simulations indicate that the majority of transmission after mass treatment will probably arise from infected individuals who have not participated in the intervention rather than from treated individuals (results not shown).

If mass treatment becomes more widely used and approved by WHO, control agencies will need to decide how the intervention would fit into a wider control programme, and at what phase of the programme it would be most useful. In all cases antimalarials to be used for mass treatment should be very carefully selected to minimize the risk of increasing drug resistance. Our results suggest that in the longer term, mass treatment may be most helpful in areas where transmission is relatively low or can be first brought to a lower level by vector control. Where there are resources to undertake repeated rounds of mass treatment, a sustained impact could also be achieved in moderate-to-high transmission settings, but the benefits of only one round of treatment can be very quickly lost in higher transmission areas in the absence of further intervention. Simulations such as the ones presented here can provide some approximate estimates of the impact of mass treatment in specific settings which can then be tested further in the field. Our results demonstrate the importance of taking local transmission conditions into account and considering long-term investment in the intervention in order to make mass treatment a successful component of a control strategy.

## Supporting Information

Text S1 **Further methods and result.**
(DOC)
