# Supplementary material for: The Potential Contribution of Mass Treatment to the Control of Plasmodium falciparum Malaria
Source: PLoS One. 2011 May 24;6(5):e20179. doi: 10.1371/journal.pone.0020179 (PMC3101232; doi:10.1371/journal.pone.0020179)
Supplement: Text S1 — Further methods and result. (DOC) [file pone.0020179.s001.doc]

The potential contribution of mass treatment to control of *P. falciparum* malaria

Supplementary Information: Further methods and results

LC Okell^1^, JT Griffin^1^, I Kleinschmidt^2^, TD Hollingsworth^1^, TS Churcher^1^, MJ White^1^, T Bousema^3^, CJ Drakeley^3^, AC Ghani^1^

^1^ MRC Centre for Outbreak Analysis & Modeling, Department of Infectious Disease Epidemiology, Imperial College London, UK.

^2^ Department of Epidemiology & Population Health, London School of Hygiene & Tropical Medicine, UK.

^3^ Department of Infectious & Tropical Diseases, London School of Hygiene & Tropical Medicine, UK.

**Transmission model**

An overview of the model structure is shown in Figure S1. The model is as previously described in with a few alterations. Additional or altered parameters are given in Table 1 in the main text. We consider a longer duration of infectivity after treatment with certain antimalarials based on recent analysis . Since patients remained gametocytaemic by molecular analysis even after successful treatment which cleared asexual parasitaemia according to standard microscopy, we assumed treated individuals would be infectious but slide-negative for a time after treatment. We also allowed for superinfection in treated individuals who have not yet cleared parasites, discounting the proportion who would be protected by effective antimalarials, approximated as the duration of prophylaxis provided by a drug divided by the duration of the treated infectious state under that drug regimen. Where the duration of prophylaxis was longer than the treated infectious state we assumed zero probability of superinfection.

**Figure S1 (next page).** Overview of the model structure in the human population for a single age and exposure group. Humans are classified into one of 6 states: susceptible and uninfected (S), asymptomatic and detectable by microscopy (A), asymptomatic and undetectable by microscopy (U), symptomatic and untreated (D), symptomatic and treated (T) and protected by prophylaxis (P). Bold arrows indicate the movement between states following a mass drug administration (MDA). For a mass screen and treat (MSAT) programme using microscopy screening, those in the undetectable U state and the susceptible S state would not be treated. For an MSAT programme using PCR screening, those in the susceptible S state would not be treated. The infectiousness of an individual following treatment is determined by the drug type and by their infection state prior to treatment. The mosquito population is also modeled dynamically (not shown, see Methods, main text).

**Comparison with trial data**

We reviewed literature to identify studies measuring the impact of mass treatment for comparison with model output. We used a previous systematic review and additionally searched PubMed with the terms “mass treatment malaria” and “active case detection malaria”. Our inclusion criteria were (i) description of a control group (>1 village) and (ii) no use of vector control in addition to mass treatment, since it would be difficult to distinguish the impact of the two interventions in the data. We found two studies matching these criteria , and chose to compare our model to that with the most detailed data available, which was a field trial of MDA in Burkina Faso in 1960-1 .

Trial details are as described in the main text. Additional details are that MDA was carried out from June to December 1960, with slide-prevalence surveys among children under 9 years carried out every 28 days during this period, and at one further follow up in March 1961. We set the coverage of MDA to be the average reported in the paper for each trial arm (92% for the fortnightly MDA group and 85% for the monthly MDA group). The authors report a low level of individual behavioural consistency in participation in successive MDA rounds and therefore we assumed individual correlation in attendance was 0.25. All individuals were given a single dose of either chloroquine-primaquine or amodiaquine-primaquine. The efficacy of this regimen at the time of the study was not certain, given lack of PCR confirmation of recrudescence versus reinfection. However, in a previous publication by the same authors in the same area , 15 out of 19 children (79%) of children aged 0-9 years were cured of parasitaemia 4 days after 1-2 doses of chloroquine. We tried simulations assuming the 1-dose regimen had an efficacy of 60% and 95% for sensitivity analysis. We assumed the duration of infectivity after treatment would have been similar as for other non-ACT (Table 1, main text) if chloroquine and amodiaquine had been used alone. However since primaquine was also in the treatment regimen we allowed for a further reduction in the duration of infectivity (80.6% see Table 1, main text). The dose of primaquine used (15mg per adult) was only about 40% of the dose used in the clinical trial from which we obtained parameters for the duration of infectivity after treatment . Therefore for sensitivity analysis we also tried assuming the size of the primaquine effect would be only 40% of the size, i.e. approximately 30% reduction in the duration of infectivity. We assumed pregnant women were treated during the original trial, as there is no report of exclusion. We also assumed low availability of other malaria control interventions given the time and area in which the trial took place, with no insecticide-treated bednets and only 1% of symptomatic cases receiving antimalarial treatment.

We fitted our model by Poisson maximum likelihood to the data on the entomological inoculation rate (EIR) in adults in control villages which was measured from January to December 1960 (Figure S2). Children are assumed to have lower exposure to vector biting. We modeled *Anopheles gambiae s.l.* and *An. funestus* populations separately, and to fit the data we varied the annual average mosquito density and parameters describing the seasonal variation in the vector birth rate, as described previously . In the data, the *An. gambiae* *s.l.* vectors were not speciated so we assumed 100% *An. gambiae s.s.*, which is predominant in the area . Since we do not consider any vector control in the simulation of this trial data, and fit directly to the EIR, the model is robust to the assumed species. We present the average results of 10 stochastic model realizations and also the range of results from 20 simulations. The population sizes simulated were as in the trial data for each arm (see main text). The total population size of the control villages was not reported, so we assumed 2000 individuals based on the number of children present in the surveys.

In the trial data, slide-prevalence of infection among children aged 2-10 at baseline was 30-60%. Before the start of the intervention, the control villages had a higher prevalence than in the intervention villages receiving fortnightly MDA (Figure S3A), but were comparable with the intervention villages receiving MDA every 28 days (Figure S3B). It is not clear if this resulted from a long-term difference in transmission intensity between control and intervention villages or chance variation. During MDA, prevalence reduced substantially in both intervention groups but 3 months after the end of MDA, prevalence rose rapidly and reached a level close to that in control groups. Based on the reported EIR, the model prediction of slide-prevalence was higher than observed in the data in all trial arms (Figures S3A & B). The model was previously fitted to a large number of paired EIR and prevalence data points but there is variability in the relationship between these measures across age groups and geographic sites and EIR measurements are imprecise. Therefore we also ran simulations in which we reduced average annual mosquito densities (keeping the seasonal pattern and the ratio of *An. gambiae* to *An. funestus* constant) to match the observed prevalence in the control villages. This matched the prevalence data better at the pre-intervention measure and during the MDA intervention, although worsened the fit at the follow up measure (Figures S3C & D). Assuming a lower efficacy of treatment further improved the model prediction (Figures S3E & F, as shown in the main text). Varying the effect of primaquine as described above had negligible effect on the predictions (results not shown). None of the simulations were able to match the observed prevalence 3 months after the end of MDA, which rose far more rapidly than predicted in the MDA villages (see Discussion, main text). However EIR data were lacking for this time period and control prevalence was also higher than predicted based on the EIR in the year before, suggesting that the annual EIR in the second season may have been higher than in the first season.

**Figure S2** Comparison of model output with trial data: EIR from *An. funestus* and *An. gambiae* in the control villages during the time period of the MDA intervention and model-fitted curves.

**Figure S3.** Comparison of model output with trial data: % slide-prevalence in 2-10 year olds (*Pf*PR2-10) and model-predictions under different assumptions. Panels on the left show MDA villages which received treatment every 14 days and panels on the right, villages receiving treatment every 28 days (A) & (B) model predictions based on observed EIR, assuming 95% treatment efficacy; (C) & (D) model predictions based on mosquito densities scaled to match the prevalence in control villages, assuming 95% treatment efficacy; (E) & (F) as (C) & (D) but assuming 60% treatment efficacy.

**
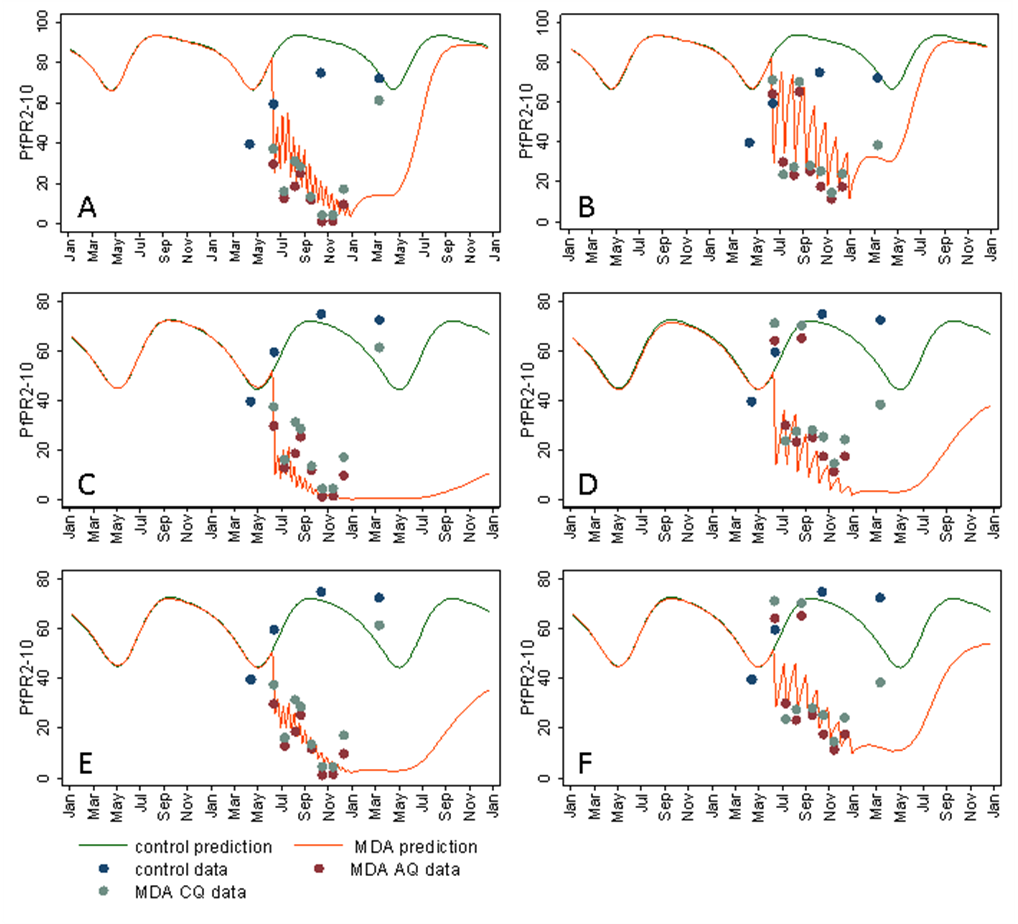
**

**Pregnancy prevalence**

Assuming that pregnant women may be excluded from a mass treatment programme, we estimated the prevalence of pregnancy as follows. We set the total fertility rate (the average number of live births per woman during her child-bearing years, defined as age 15-45) as the average of the total fertility rates in the most recent Demographic and Health Surveys (DHS) for the Sub-Saharan African region. Data were available for 25 countries. We also allowed for miscarriages and abortions as described in : the abortion rate has been estimated at a rate of 29 per 1000 women per year in Africa , and the rate of miscarriage is estimated as 20% of live births plus 10% of abortions. The length of time spent pregnant between the ages of 15 and 45 was estimated as the number of live births multiplied by 9 months, and the number of miscarriages and abortions multiplied by 2 months. For simplicity we assumed the time spent pregnant was evenly distributed between the ages of 15-45 years, resulting in a figure of 14.2% prevalence of pregnancy among women in this age group. Coverage of mass treatment was then adjusted to exclude 7.1% of 15-45 year olds, assuming 50% of the population is male.

**Antimalarial effects on transmission**

Parameter values are given in the main text, Table 1. Estimates of the duration of infectivity of human cases to mosquitoes after treatment with different antimalarials was based on analysis of gametocytaemia measured in a clinical trial . Probabilities of transmission to mosquito after different treatments were based on blood-feeding experiments where available , or data on gametocyte density .

Antimalarial regimens which act primarily against asexual blood stages, such as sulphadoxine-pyrimethamine-amodiaquine (SP-AQ), are assumed to reduce the duration of infectivity to 56 days . The probability of transmission to mosquitoes after treatment relative to those who are untreated was estimated from a trial among asymptomatic cases . This study found a log area under the curve of gametocyte density that was approximately 70% lower during 56 days of follow up among those receiving SP treatment relative to placebo. For this calculation we assumed infectivity to mosquitoes is proportional to log-gametocyte density and that infectivity is constant over time. Combination therapies containing artemisinin (ACT) which have gametocytocidal action reduce the duration of infectivity to 13.4 days and the level of infectiousness by a further 32% compared to non-gametocytocidal regimens . In a different trial, the addition of primaquine (PQ) to ACT reduced the duration of infection by 80.6% compared to ACT alone , so we estimated 13.4 days x (1-0.806) = approximately 3 days. We conservatively assumed the probability of transmission to mosquitoes would be the same after ACT-PQ as after ACT treatment despite the shorter duration of infectiousness. Absolute values vary between studies, so when combining results of different studies we assume the same relative value between parameters rather than the measured values where necessary.

We also compared the use of long-acting treatments such as SP, where the period of prophylaxis is around 30 days in the absence of significant parasite resistance with shorter acting treatments such as artemether-lumefantrine which has a prophylactic time of around 10 days .

**Vector control**

To simulate vector control, we modeled the use of long-lasting insecticide-treated nets (LLIN) as previously described . We assumed ITN were given out as mass campaigns at regular intervals, with a correlation of 0.5 in the probability of an individual receiving an LLIN over successive distribution rounds. Use and efficacy of LLIN decline following distribution in the model as has been observed in endemic settings. Distribution in each mass campaign is staggered over time to allow for the delay involved in reaching a larger population with the intervention. For all simulations in the results section “Potential for elimination”, we assume a baseline low coverage of LLIN at 20% introduced 10 years ago to represent the current situation in many endemic countries. This has the effect that for a given EIR, slide-prevalence of infection is 13%-45% lower, with the percentage reduction being greatest in the lower transmission settings (see Figure S4A). We also explored the scenario that LLIN were rapidly scaled up from 20% coverage at baseline to 80% coverage, which has the effect of gradually reducing transmission over several years. At 2 years after the intervention, slide-prevalence is reduced 34-50% by this control measure (Figure S4B). We combined this intervention with mass treatment, with results as described in the main text.

**Figure S4.** The impact of vector control on slide-prevalence over time in scenarios of varying initial transmission levels. (A) LLIN are introduced at 20% coverage at year 0, with staggered mass campaigns repeated every 5 years at the same coverage. (B) LLIN are at 20% coverage at baseline, then rapidly scaled up to 80% coverage at year 0 with repeated campaigns at 80% coverage every five years.

**References**
